# Health gains and financial protection from human papillomavirus vaccination in Ethiopia: findings from a modelling study

**DOI:** 10.1093/heapol/czab052

**Published:** 2021-05-04

**Authors:** Allison Portnoy, Steven Sweet, Dawit Desalegn, Solomon Tessema Memirie, Jane J Kim, Stéphane Verguet

**Affiliations:** Center for Health Decision Science, Harvard T.H. Chan School of Public Health, 718 Huntington Avenue, 2nd Floor, Boston, MA 02115, USA; Center for Health Decision Science, Harvard T.H. Chan School of Public Health, 718 Huntington Avenue, 2nd Floor, Boston, MA 02115, USA; Vitalant Research Institute, 270 Masonic Avenue, San Francisco, CA 94118, USA; Department of Gynecology and Obstetrics, College of Health Sciences, Addis Ababa University, Addis Ababa, Ethiopia; Department of Pediatrics and Child Health, College of Health Sciences, Addis Ababa University, Addis Ababa, Ethiopia; Center for Health Decision Science, Harvard T.H. Chan School of Public Health, 718 Huntington Avenue, 2nd Floor, Boston, MA 02115, USA; Center for Health Decision Science, Harvard T.H. Chan School of Public Health, 718 Huntington Avenue, 2nd Floor, Boston, MA 02115, USA; Department of Global Health and Population, Harvard T.H. Chan School of Public Health, 665 Huntington Avenue, Boston, MA 02115, USA

**Keywords:** Extended cost--effectiveness analysis, cervical cancer, human papillomavirus, vaccination, equity, Ethiopia

## Abstract

High out-of-pocket (OOP) medical expenses for cervical cancer (CC) can lead to catastrophic health expenditures (CHEs) and medical impoverishment in many low-resource settings. There are 32 million women at risk for CC in Ethiopia, where CC screening is extremely limited. An evaluation of the population health and financial risk protection benefits, and their distributional consequences across socioeconomic groups, from human papillomavirus (HPV) vaccination will be critical to support CC prevention efforts in this setting. We used a static cohort model that captures the main features of HPV vaccines and population demographics to project health and economic outcomes associated with routine HPV vaccination in Ethiopia. Health outcomes included the number of CC cases, and costs included vaccination and operational costs in 2015 US dollars over the years 2019–2118 and CC treatment costs over the lifetimes of cohorts eligible for vaccination in Ethiopia. We estimated the household OOP medical expenditures averted (assuming 68% of direct medical expenditures were financed OOP) and cases of CHE averted. A case of CHE was defined as 40% of household consumption expenditures, and the cases of CHE averted depended on wealth quintile, disease incidence, healthcare use and OOP payments. Our analysis shows that, assuming 100% vaccine efficacy against HPV-16/18 and 50% vaccination coverage, routine HPV vaccination could avert up to 970 000 cases of CC between 2019 and 2118, which translates to ∼932 000 lives saved. Additionally, routine HPV vaccination could avert 33 900 cases of CHE. Approximately one-third of health benefits would accrue to the poorest wealth quintile, whereas 50% of financial risk protection benefits would accrue to this quintile. HPV vaccination can reduce disparities in CC incidence, mortality and household health expenditures. This understanding and our findings can help policymakers in decisions regarding targeted CC control efforts and investment in a routine HPV vaccination programme following an initial catch-up programme.

Key messages

- We used a static cohort model that captures the main features of HPV vaccines and population demographics to project health and economic outcomes, including financial risk protection and distributional impact, associated with routine HPV vaccination in Ethiopia.
- Assuming 100% vaccine efficacy against HPV-16/18 and 50% vaccination coverage, our analysis shows that routine two-dose HPV vaccination could avert up to 970 000 cases of cervical cancer between 2019 and 2118, which translates to ∼932 000 lives saved.
- Approximately one-third of health benefits would accrue to the poorest wealth quintile, whereas 50% of financial risk protection benefits would accrue to this quintile.
- This analysis can help make decisions regarding routine HPV vaccination, including how to progress towards cervical cancer elimination both efficiently and equitably.

## Introduction

Ethiopia is a large (population of 109 million in 2018), low-income country (per capita gross domestic product of $770 in 2018) in East Africa, with 24% of the population living below the national poverty line (World Bank, 2019). Ethiopia is also very resource constrained with annual per capita health expenditures of $28 (World Bank, 2019), compared to $70 in neighbouring Kenya or $46 in Uganda, and more than one-third of health expenditures in Ethiopia is financed by out-of-pocket (OOP) payments (World Bank, 2019). Cervical cancer (CC) is the second leading cause of female cancer deaths in this setting (Memirie *et al.*, 2018) and could be among the top 20 causes of medical impoverishment (Verguet *et al.*, 2016b). While the CC incidence rate in Ethiopia is lower compared to neighbouring Kenya or Uganda, the number of women over the age of 15 years at risk for CC is more than double at 32 million, but only 2.4% of patients have access to radiotherapy to treat CC (Datta *et al.*, 2014). The disease burden attributable to CC and other human papillomavirus (HPV)-induced anogenital and oropharyngeal cancers is largely preventable with HPV vaccination, but the uptake of HPV vaccines remains low. Currently, Ethiopia has administered HPV vaccination to a single cohort of 14-year-old girls in 2018–19 with ∼95% coverage (Memirie, 2020). However, there are no additional plans to implement routine HPV vaccination, due to restrictions from the current global vaccine shortage (World Health Organization, 2018).

CC treatment can create a large financial burden on households, with direct medical costs of ∼$330 per patient for consultations, investigations and drugs (Hailu and Mariam, 2013), without counting for the additional burden of costs such as time losses and transportation expenses. Public financing of HPV vaccination has the potential to increase the uptake of the vaccine and decrease mortality due to CC but also can eliminate the incidence of these potentially large OOP expenditures associated with care-seeking for CC treatment. Increased HPV vaccination coverage can improve the distribution of both health and financial outcomes in populations (Levin *et al.*, 2015), and it can reduce HPV-related hospitalizations, prevent HPV-related impoverishment and bring significant cost savings and financial risk protection (FRP) benefits to the affected poor women and their households (Kim and Goldie, 2008; Campos *et al.*, 2017; Levin *et al.*, 2015). Public finance of HPV vaccination therefore contributes to reaching the Sustainable Development Goals (SDGs) 1 and 3 to ‘end poverty in all its forms everywhere’ and to ‘achieve universal health coverage, including financial risk protection for all’, respectively (United Nations, 2015) and to the World Health Organization’s global call to action for the elimination of CC, including increased HPV vaccination (World Health Organization, 2019).

Beyond vaccinating the initial cohort of 14-year-old girls (in 2018–19), an evaluation of continued annual routine HPV vaccination in terms of preventing both CC cases and deaths and medical impoverishment from OOP health-related expenditures will be critical to support CC prevention in Ethiopia. Traditional economic evaluations such as cost-effectiveness analysis (CEA) of health interventions often only account for total health impacts and costs incurred (Jamison, 2009), neglecting the potentially significant benefits accruing beyond this narrow scope, such as important equity and distributional considerations (Barnighausen *et al.*, 2014; Bloom *et al.*, 2018). As decision-makers evaluate HPV vaccination, the ability to demonstrate its broader economic impact will be critical. One possibility is to use extended cost-effectiveness analysis (ECEA) methods (Verguet *et al.*, 2016a; 2013; 2015a,b), which can complement traditional CEA in assessing the equity, FRP and distributional (e.g. across socioeconomic or income groups) components of HPV vaccination programmes. Such considerations for reducing health disparities arising from socioeconomic inequalities and reducing financial consequences of ill health are recommended criteria for priority setting in the health sector (Norheim *et al.*, 2014). ECEA therefore can help document performance along the dimensions of both efficiency and equity, towards tracking progress with respect to SDGs 1 and 3. The objective of this analysis is therefore to evaluate the potential health, equity and FRP benefits of sustaining routine HPV vaccination in Ethiopia.

## Methods

We conducted an economic evaluation of routine HPV vaccination by quantifying the health, FRP and distributional benefits of rolling out HPV vaccination nationally in Ethiopia, consistent with the dashboard of health and financial outcomes reported in ECEA (Verguet *et al.*, 2016a).

### General approach

We chose the health sector perspective to evaluate a hypothetical programme for routine HPV vaccination in Ethiopia. In addition to health benefits (in this case, CC cases and deaths averted), we estimate intervention impact along three dimensions: (1) household OOP medical expenditures averted by HPV vaccination, (2) FRP benefits provided through the reduction of OOP medical expenditures by HPV vaccination and (3) distributional impact across the wealth strata (e.g. income quintiles) of the population (Verguet *et al.*, 2016a).

### Disease modelling—CC cases

We used a previously validated static cohort model approach to estimate the number of CC cases and deaths averted associated with routine HPV vaccination in Ethiopia (Goldie *et al.*, 2008; Lee *et al.*, 2013; Campos *et al.*, 2017; Ozawa *et al.*, 2017; Brisson *et al.*, 2020; Burger *et al.*, 2020; Canfell *et al.*, 2020). The model tracks a cohort of girls starting at a target age (e.g. 9 or 14 years) through their lifetimes, comparing health and cost outcomes with and without HPV vaccination. The population-based model uses data on the age-specific incidence of CC (Memirie *et al.*, 2018), HPV-16/18 type distribution in CC (Bekele *et al.*, 2010) and demographics (e.g. population size and background age-specific mortality rates) to estimate reductions in CC incidence over time (United Nations Population Division, 2019). We applied these age-specific cancer incidence reductions to the baseline age-specific cancer incidence rates in Ethiopia (Supplementary Appendix Table A1), in order to estimate the number of CC cases averted associated with HPV vaccination, adjusting for population growth over time. We stratified the CC cases into quintiles by using the distribution of sexually transmitted disease (STD) prevalence (i.e. 31%, 7%, 8%, 10% and 43%, from poorest to richest quintile, respectively) from the Ethiopian Demographic and Health Survey (DHS) as a proxy for CC prevalence, given that HPV, a sexually transmitted infection, is the main cause of CC and there has been shown to be an increased risk of HPV with a pre-history of STD (Muñoz *et al.*, 2003; Central Statistical Agency [Ethiopia] and ICF, 2016, Belayneh *et al.*, 2019). We also tested alternative distributions of CC cases in sensitivity analyses.

### Vaccination scenarios

We conducted analyses to evaluate the impact of annual, routine (i.e. fixed facility delivery) two-dose HPV vaccination of 9-year-old girls at an average of 50% coverage. We used the coverage wealth-gradient for diphtheria-tetanus-pertussis third dose (DTP3) coverage from the DHS as a proxy to apportion aggregate 50% coverage by household wealth quintile, resulting in the following coverage levels from poorest to richest quintile: 26%, 48%, 50%, 52% and 74% (i.e. the ‘50% coverage gradient’ scenario) (Central Statistical Agency [Ethiopia] and ICF, 2016). We assumed 100% protection against HPV-16 and HPV-18 infections over the lifetime of vaccinees for a two-dose vaccination schedule. In scenario analysis, we assumed a flat 50% vaccination coverage (i.e. the ‘50% flat coverage’ scenario across all wealth quintiles). CC cases (and costs) averted were calculated in comparison with a strategy of no HPV vaccination (i.e. Ethiopia’s status quo and without the 2018–19 single-age cohort vaccinated).

### CC costs

CC treatment costs included direct medical costs for cancer staging, treatment, palliative care and follow-up associated with stage-specific International Federation of Gynecology and Obstetrics treatment protocols, assuming that cancer treatment costs were not dependent upon vaccination coverage level (Campos *et al.*, 2016; 2017). We assumed the stage distribution of detected cancers to be 9% Stage I, 36% Stage II, 47% Stage III and 8% Stage IV (Canfell *et al.*, 2020). We assumed country-specific 5-year survival for CC cases by stage, validated against age-specific mortality rates (Bray *et al.*, 2018; Canfell *et al.*, 2020). We assumed that all cancer staging, treatment, palliative care and follow-up took place at a tertiary facility. To estimate the unit cost of each procedure, we identified available data from the published literature (Berraho *et al.*, 2012; Campos *et al.*, 2015; Goldie *et al.*, 2005; Katanyoo *et al.*, 2014; Ranson and John, 2002; Shi *et al.*, 2012; Termrungruanglert *et al.*, 2012) and unpublished data (Constenla *et al.*, 2008). All costs were converted to 2015 US dollars using local consumer price index deflators and exchange rates (World Bank, 2019). To extrapolate published estimates for CC treatment costs from their original settings, accounting for variation in income level, we adjusted unit costs using an index of tertiary inpatient visit costs from WHO-CHOICE (World Bank, 2019; World Health Organization, 2008), resulting in about $700 provider cost for the CC treatment cost (per person) in Ethiopia in 2015 US dollars (Campos *et al.*, 2016).

### Vaccination costs

We assumed a vaccine cost of $4.50 per dose for HPV vaccine (Gavi, the Vaccine Alliance, 2017), which corresponds to the subsidized cost of HPV vaccine procured by Gavi, the Vaccine Alliance, for low-income countries such as Ethiopia. We also included costs for vaccine wastage at a rate of 5% for a single-dose vial, liquid formulation (Parmar *et al.*, 2010). Lastly, we assumed a one-time introduction cost of $2.00 per girl in the first year of the vaccination programme, followed by a recurrent delivery cost of $1.70 per girl, including costs for personnel, training, social mobilization, disease surveillance, programme management and other recurrent costs (Botwright *et al.*, 2017; Gavi, the Vaccine Alliance, 2013).

### Financial risk protection

Household medical expenditures related to the treatment of CC cases can be averted with roll-out of HPV vaccination that reduces the incidence of CC cases. Such household OOP direct medical costs averted would then depend on the number of CC cases prevented, probability of seeking care (for CC) and cost of health care. Sixty-eight percent of health expenditures associated with non-communicable diseases are financed by OOP payments in Ethiopia (Federal Democratic Republic of Ethiopia Ministry of Health, 2018). Therefore, we assumed that an individual’s OOP burden for treating CC would be ∼68% of the total health sector treatment cost for CC, and the government would cover the remaining 32% of the costs, in the base case. The base-case OOP payments for CC treatment in Ethiopia were then assumed to be $482 (i.e. 68% of $700).

By averting OOP expenditures related to the treatment of CC cases, roll-out of HPV vaccination then provides FRP benefits to the population. Two metrics of (lack of) FRP are commonly used by the World Bank and the World Health Organization in tracking progress towards universal health coverage: catastrophic health expenditures (CHEs), which count the number of occurrences when OOP direct medical expenditures surpass a certain threshold of total consumption expenditures, and impoverishing health expenditures (IHEs), which count the number of occurrences when OOP direct medical expenditures push individuals/households below a defined poverty line (e.g. international poverty line of $1.90 per day, purchasing power parity) (Wagstaff *et al.*, 2018a,b).

Here, we quantify the number of cases of CHE that would be averted, counting the number of occurrences when OOP payments tied to direct medical costs of CC treatment would no longer surpass a certain threshold of total household consumption expenditures (Wagstaff *et al.*, 2018a). According to survey data, the level of household consumption expenditures by quintile in Ethiopia are $218 in the poorest quintile, $367 in the poorer quintile, $501 in the middle quintile, $682 in the richer quintile and $1418 in the richest quintile (Central Statistical Agency [Ethiopia], 2018). In order to estimate the number of cases of CHE averted, for each quintile, we compared the household expenditures for CC treatment costs to these consumption expenditure quintiles. We assumed a 40% threshold for CHE in the base case. Under this definition of CHE at a 40% threshold, CC treatment would be considered catastrophic for the poorest, poorer, middle and richer quintiles. We also used the average access proportion for radiotherapy, a key component of cancer management, across low-income countries (8.5%) (Datta *et al.*, 2014), as a proxy for CC care-seeking, which accounts for both barriers to access and other barriers to seeking care (Woldeamanuel *et al.*, 2013). We stratified this proportion into the associated consumption expenditure quintiles using the relative care-seeking percentages for STDs in the Ethiopia DHS (Central Statistical Agency [Ethiopia] and ICF, 2016), resulting in 5.5% care-seeking in the poorest quintile, 6.7% in the poorer quintile, 4.6% in the middle quintile, 9.4% in the richer quintile and 12.3% in the richest quintile.

### Analysis outcomes

The primary financial outcomes of the analysis included household OOP expenditures and cases of CHE averted related to the prevention of CC cases by quintile. Health outcomes included the number of CC cases and associated deaths averted. We discounted future costs at a rate of 3% annually. Model outcomes were aggregated over multiple birth cohorts to capture the lifetime costs and benefits of girls aged 9 years between 2019 and 2118 (i.e. 100 cohorts).

### Scenario analysis

We analysed six extensions to the base-case scenario: (1) changes in the vaccine price, (2) changes to the level of care-seeking, (3) changes to the level of OOP payments by quintile, (4) changes to the distribution of CC incidence by quintile, (5) changes to the CHE threshold and (6) changes to the distribution of income by quintile. We also examined alternative time horizons of 10, 25 and 50 years in addition to the 100-year base case.

In the first scenario analysis, we used a vaccine price of $0.20 per dose, as this is the co-financing level offered by Gavi, to introduce new vaccines in low-income countries such as Ethiopia (Gavi, the Vaccine Alliance, 2015). In the second scenario analysis, we assumed two alternatives for the proportion of CC cases that would seek care, compared to 8.5% in the base case: (1) 2.4% as a conservative lower bound, according to the estimate for access to radiotherapy in Ethiopia, and (2) 50% as an aspirational upper bound, according to the treatment access that has been achieved in upper middle-income countries (Datta *et al.*, 2014). In the third scenario analysis, we distributed OOP payments by wealth quintile following a log-normal distribution, given a shape parameter of the Gini coefficient for Ethiopia (0.35 in 2015; World Bank, 2019) to account for variations in ability to pay across socioeconomic groups: $112 for poorest, $253 for poorer, $399 for middle, $593 for richer and $1049 for richest. For this scenario analysis, CC treatment is then considered catastrophic for all quintiles at a 40% threshold. In the fourth scenario analysis, we assumed no gradient in CC incidence by quintile as well as a linear gradient: 33% of CC incidence experienced by the poorest, 27% poorer, 20% middle, 13% richer and 7% richest. In the fifth scenario analysis, we examined 10% and 25% CHE thresholds (instead of 40%). In the sixth scenario analysis, we distributed average income by wealth quintile following a log-normal distribution, given a shape parameter of the Gini coefficient for Ethiopia. We projected the Gini coefficient over time using the historical trend of available Gini coefficient estimates from the World Bank development indicators (World Bank, 2019).

### Sensitivity analysis

Four key parameters were identified for probabilistic sensitivity analysis (PSA): HPV-16/18 type distribution, age-specific CC incidence, stage distribution of CC, and stage-specific 5-year survival and treatment access (as a combined parameter). Each parameter was assigned a beta-PERT distribution for probabilistic sampling, with the bounds determined by (1) empirical data for type distribution, (2) confidence intervals estimated from CC cases in Globocan 2020 (Ferlay *et al.*, 2018), (3) assumed ±10% bounds from the base case for stage distribution and (4) assumed ±10% bounds from the base case for stage-specific probability of death following 5-year survival, if this estimate is contained between zero and one (Supplementary Appendix Table A2). Two hundred independent parameter sets were drawn, and 95% uncertainty intervals (UIs) were extracted from the 2.5 and 97.5 percentiles of the sample results obtained.

## Results

### Health outcomes of HPV vaccine

**Figure 1.**
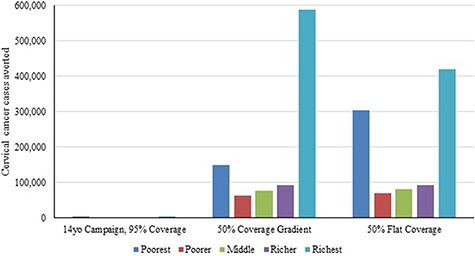
Cervical cancer cases averted over the years 2019–2118 by vaccination scenario and consumption quintile, assuming a lifelong duration of protection against HPV-16/18 infections.

Routine HPV vaccination assuming a lifelong duration of protection against HPV-16/18 infections could avert 970 000 (95% UI: 740 000–994 000) cases of CC of cohorts vaccinated over 2019–2118 in the base-case scenario (i.e. 50% coverage and 100% vaccine efficacy), compared to a strategy of no HPV vaccination. This translates to ∼932 000 (704 000–965 000) lives saved. Assuming 50% flat coverage, 31% of these health benefits would accrue to the poorest quintile compared with 43% to the richest quintile, whereas, assuming a coverage gradient averaging 50%, 15% of the health benefits would accrue to the poorest quintile compared with 61% in the richest (Figure 1). Current HPV vaccination in Ethiopia of 14-year-old girls at ∼95% coverage could avert 10 800 (8210–11 100) cases of CC, which could be extended to 54 500 (41 500–56 000) cases of CC averted if the recommended multi-age catch-up cohort of 10- to 14-year-old girls received vaccination in the first year.

### Financial outcomes of HPV vaccine

The total vaccine-related costs associated with the base-case scenario of 50% routine coverage were nearly $265 million (discounted net present value) between 2019 and 2118, but resulted in long-term cost offsets from future averted CC cases of $93 million ($71–96 million) or 41% of CC treatment costs compared with no vaccination. The vaccination programme of a single cohort of 14-year-old girls at 95% coverage exceeded $16 million but would avert $1.9 million ($1.4–2.0 million) associated with CC treatment costs (i.e. 1% of total costs compared to no vaccination).

### Financial risk protection benefits of HPV vaccine

**Figure 2.**
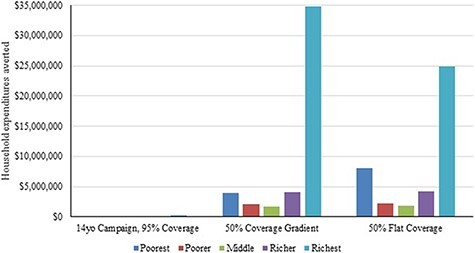
OOP expenditures averted by vaccination scenario and consumption quintile. Note: The findings assume a $482 OOP payment for cervical cancer treatment.

Assuming 100% efficacy against HPV-16/18 infections and a lifelong duration of protection, our analysis shows that routine two-dose HPV vaccination could avert $41 200 000 ($31 400 000–42 200 000; flat coverage of 50%) to $46 600 000 ($35 600 000–47 800 000; coverage gradient of 50%) in total OOP expenditures over 2019–2118 compared to no vaccination, with the bottom two quintiles accounting for ∼25% of all OOP expenditures averted in the latter (Figure 2). In terms of cases of CHE averted between 2019 and 2118, routine two-dose HPV vaccination could avert 24 600 (18 700–25 200) cases of CHE at 50% coverage gradient and 33 900 (25 800–34 700) cases of CHE at 50% flat coverage (Table 1). When examining the FRP benefits by wealth quintile, ∼33–50% of these FRP benefits (assuming a coverage gradient or flat coverage, respectively) would be experienced by the poorest quintile. In summary, per government budget expenditure, routine two-dose HPV vaccination of 9-year-old girls could avert up to 264 (193–292) CC deaths and 55 (41–56) cases of associated CHE per $100 000 spent.

**Table 1.** Cases of CHE averted by routine two-dose HPV vaccination scenario in Ethiopia, assuming a lifelong duration of protection against HPV-16/18 infections (95% uncertainty intervals in parentheses)

Scenario	Poorest	Poorer	Middle	Richer	Richest	Total
14-Year-old cohort, 95% coverage	190 (140–190)	50 (40–50)	40 (30–40)	100 (70–100)	0	380 (280–380)
10- to 14-Year-old cohorts, 95% coverage	900 (700–900)	260 (200–260)	210 (160–210)	490 (370–490)	0	1860 (1430–1860)
Routine, 50% coverage gradient	8200 (6200–8400)	4250 (3240–4360)	3530 (2700–3620)	8600 (6560–8810)	0	24 600 (18 700–25 200)
Routine, 50% flat coverage	16 700 (12 700–17 100)	4690 (3580–4800)	3740 (2860–3830)	8760 (6680–8970)	0	33 900 (25 800–34 700)

The findings assume a $482 OOP payment for cervical cancer treatment, which is considered a case of CHE for the poorest, poorer, middle and richer consumption quintiles at a 40% threshold. Estimates were rounded to three significant figures, or the nearest ten, depending on magnitude.

**Figure 3.**
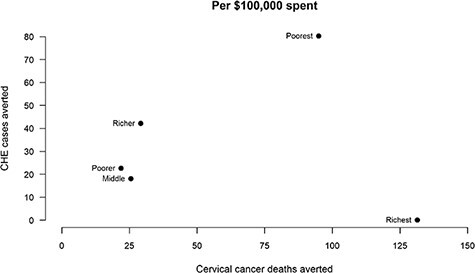
Cervical cancer deaths and cases of CHE averted by routine two-dose HPV vaccination in Ethiopia, as per government budget expenditure.

The number of CC deaths and CHE cases averted for the base-case scenario, assuming flat 50% coverage with a vaccine that is 100% efficacious against HPV-16/18 infections, is shown in Figure 3. While the combination of disease incidence and care-seeking by quintile yields the greatest number of deaths averted for the richest quintile, the poorest quintile would experience the greater FRP benefits at 80 (61–82) cases of CHE averted per $100 000 spent.

### Scenario analysis

In the first scenario analysis, if Ethiopia were to introduce the HPV vaccine with Gavi support and face a vaccine price of $0.20 per dose (rather than $4.50 per dose as in the base case), the $265 million in vaccination programme costs would substantially be reduced to $37 million (discounted at 3% per annum), respectively. Given that $93 million ($71–96 million) in CC treatment costs would be averted in the routine programme at 50% coverage, a routine vaccine programme would be cost-saving at this reduced vaccine price. In the second scenario analysis, if our assumed proxy for care-seeking percentage were decreased from 8.5% in the base case to 2.4%, the number of CHE cases averted would decrease, with routine two-dose HPV vaccination averting 6930 (5330–7140) cases of CHE (assuming a 50% coverage gradient) or 9550 (7310–9770) cases of CHE (assuming 50% flat coverage). On the other hand, if our assumed proxy for care-seeking percentage were increased from 8.5% in the base case to 50%, the number of CHE cases averted would greatly increase, as expected, with routine two-dose HPV vaccination averting 145 000 (110 000–148 000) cases of CHE (assuming a 50% coverage gradient) or 199 000 (152 000–204 000) cases of CHE (assuming 50% flat coverage) up from 24 600 (18 700–25 200) and 33 900 (25 800–34 700) cases of CHE in the base case, respectively (Supplementary Appendix Table A3). In the third scenario analysis, assuming a distribution of OOP payments by quintile, OOP expenditures averted would decrease as the bottom 60% of households would face lower OOP payments relative to the base case. However, cases of CHE averted would increase as the richest quintile also faces OOP payments that would be considered catastrophic, even with the reduced OOP payment levels. In the fourth scenario analysis, when CC incidence is evenly distributed on a linear gradient with the poorest quintile experiencing the greatest disease burden down to the richest quintile experiencing the least disease burden, the total number of cases of CHE averted would increase, with the poorest quintile experiencing nearly one-third of the FRP benefits (Supplementary Appendix Table A4). Similarly, removing the STD prevalence-derived gradient increases the total number of cases of CHE averted (Supplementary Appendix Table A5). In the fifth scenario analysis, a CHE threshold of either 10% or 25% would add the richest quintile to the group of quintiles experiencing CHE, increasing the number of cases of CHE averted by 150–300% (Table 2). In the sixth scenario analysis, projecting the income distribution, OOP payments levels and CHE cut-offs by quintile, cases of CHE averted would remain the same as OOP payments would be considered catastrophic for the poorest, poorer, middle and richer quintiles, but not the richest quintile according to these projections (Supplementary Appendix Table A6). However, total OOP expenditures averted would increase by ∼16% (assuming 50% flat coverage). Cases of CHE averted estimated based on alternative time horizons of 10, 25 and 50 years are also presented (Supplementary Appendix Table A7).

**Table 2.** Cases of CHE averted by routine two-dose HPV vaccination scenario over 2019–2118 in Ethiopia: varying the CHE threshold at 40, 25 and 10% of consumption expenditures (95% uncertainty intervals in parentheses)

Scenario	Wealth quintile					Total by CHE threshold		
	Poorest	Poorer	Middle	Richer	Richest	40%	25%	10%
14-Year-old cohort, 95% coverage	190 (140–190)	50 (40–50)	40 (30–40)	100 (70–100)	580 (440–590)	380 (280–380)	960 (720–970)	960 (720–970)
Routine, 50% coverage gradient	8200 (6200–8400)	4250 (3240–4360)	3530 (2700–3620)	8600 (6560–8810)	72 300 (55 100–74 000)	24 600 (18 700–25 200)	96 800 (73 800–99 200)	96 800 (73 800–99 200)
Routine, 50% flat coverage	16 700 (12 700–17 100)	4690 (3580–4800)	3740 (2860–3830)	8760 (6680–8970)	51 700 (39 500–53 000)	33 900 (25 800–34 700)	85 600 (65 300–87 700)	85 600 (65 300–87 700)

The findings assume a $482 OOP payment for cervical cancer treatment, which is considered a case of CHE for the poorest, poorer, middle and richer consumption quintiles at a 40% threshold. At a 10% or 25% threshold, all wealth quintiles would experience a case of CHE. Estimates were rounded to three significant figures, or the nearest ten, depending on magnitude.

## Discussion

Using a static cohort model that incorporates HPV vaccine characteristics, age-specific CC incidence and HPV type distribution, population demographics, FRP and distributional analysis, we projected that routine two-dose HPV-16/18 vaccination could avert 932 000 CC deaths and 33 900 cases of CHE over 2019–2118 in the simulated base-case scenario (50% coverage and 100% efficacy against HPV-16/18 infections) in Ethiopia. The percentage of CHE cases averted is highly sensitive to the rate of care-seeking for CC that we assumed in this population (8.5%), which is an average across low-income settings as we relied on an estimate of access to radiotherapy, and the 2.4% Ethiopia-specific estimate is likely to be an underestimate of CC care as not all CC case management services are available in tertiary facilities. However, we examined the impact of assuming a 2.4% rate of care-seeking, as well as an aspirational 50% rate of care-seeking in scenario analysis. Assuming 50% flat coverage (which could be reached over the future 2019–2118 time period), ∼30% of the health benefits from HPV vaccination would accrue to the poorest wealth quintile, whereas ∼50% of the FRP benefits would accrue to the same poorest quintile. While the combination of disease incidence and care-seeking by quintile (proxied by STD prevalence and care-seeking, respectively) would yield the greatest health benefits for the richest quintile, the magnitude of the FRP benefits experienced by the poorest quintile (in addition to their experiencing of the second largest health benefits across quintiles) indicates that routine two-dose HPV vaccination could be equity-improving overall. These results underscore the importance of considering perspectives of both total health gains and equity when evaluating public health interventions: in this particular case, we observe that HPV vaccination could be both pro-rich and pro-poor from a health benefit perspective and be pro-poor from a financial protection perspective.

Given the SDG focus on poverty reduction (United Nations, 2015) and the 24% of the population living under the national poverty line in Ethiopia (World Bank, 2019), the incorporation of equity dimensions in this analysis brings valuable contextual elements for policymaking. In the particular case of HPV vaccination, our analysis points to potentially pro-poor dimensions of HPV prevention and control in Ethiopia when viewed from an FRP perspective. However, it is important to note that our base-case analysis assumed a cost of $10.70 per girl for routine HPV vaccination (in the years following introduction), which amounts to ∼0.2% of Ethiopia’s current annual health expenditures (at 50% vaccination coverage) (World Bank, 2019). Therefore, the price of the vaccine would be an important consideration for the budgetary impact: this is why one scenario analysis examined a reduced vaccine price ($0.20 per dose), which would reduce HPV vaccination cost to ∼0.04% of current annual health expenditures.

There are several important limitations to this analysis. First, we projected intervention impact for only a single cohort of 14-year-old girls in Ethiopia (based on the previously implemented 2018–19 vaccination effort) as a comparison to the roll-out of routine HPV vaccination; even though there are plans in discussion, while there remains a global HPV vaccine shortage, it is currently not feasible for additional cohorts to be vaccinated (World Health Organization, 2018). If Ethiopia were able to vaccinate 10- to 14-year-old girls at 95% coverage prior to a routine programme, ∼54 500 CC cases and 1860 cases of CHE would be averted, as compared to 970 000 CC cases and 33 900 cases of CHE averted in the base-case scenario. Additionally, it is important to note that while the costs of vaccination are experienced in the first year of the programme, these health benefits and costs averted would accrue over the lifetime of those vaccinated.

Second, in order to stratify CC cases by wealth quintile, we assumed that CC incidence and deaths within each quintile would mimic the distribution of STD prevalence across wealth quintiles in Ethiopia (Central Statistical Agency [Ethiopia] and ICF, 2016). As a result, the STD prevalence rankings by quintile—Richest > Poorest > Richer > Middle > Poorer—largely drove the distribution in health benefits and their rankings across quintiles (particularly when combined with the coverage gradient and care-seeking proportions). Stratifying by STD prevalence is only one possible stratification approach: we would need primary data on CC incidence and mortality across socioeconomic groups to develop additional distributional estimations in order to fully validate such a stratification approach. In addition, our static cohort model does not capture dynamic effects in terms of both health and economic outcomes that could differ by wealth quintile. While we currently have no data to support these analyses, future exercises should explore the levels of transmissions and ‘social mixity’ both between and within wealth quintiles. We might expect that the level of household consumption would be correlated with health outcomes, such that richer households can afford greater expenditures on healthcare. According to Global Burden of Disease study cross-country estimates, we might also expect to see the highest CC disease burden in the poorest sociodemographic group (Naghavi *et al.*, 2017), which would then lead to an increase in the impact of HPV vaccination among the poorest individuals. Therefore, we included a scenario analysis applying a linear gradient to CC incidence by quintile in order to demonstrate what this impact might look like (Supplementary Appendix Table A4).

Third, we have only estimated FRP benefits from HPV vaccine using CHE as a metric for lack of FRP (Wagstaff *et al.*, 2018a). Other FRP measures include estimating the incidence of IHEs, i.e. the number of households for whom OOP health-related expenditures would push them below a defined poverty line (e.g. international poverty line of $1.90 per day, purchasing power parity) or the estimation of a money-metric value of insurance (Verguet *et al.*, 2016a; Wagstaff *et al.*, 2018b). Importantly, due to the lack of empirical data on OOP costs related to CC treatment, this analysis assumed OOP levels at 68% of the total treatment cost based on Ethiopia’s national health accounts (Federal Democratic Republic of Ethiopia Ministry of Health, 2018). In addition, our estimation of FRP gains did not include direct non-medical costs such as transportation, which may differ by wealth quintile and geography (79% of individuals live in rural areas in Ethiopia; World Bank, 2019), nor indirect costs (e.g. wages lost and time losses), which may significantly augment the potential FRP benefits of HPV vaccination.

Fourth, this analysis captured the costs of the vaccination programme over 100 years; we assumed that CC incidence rates would be stable over this time period. Likewise, we assumed constant expenditures across this time period and no changes to the distribution of income by wealth quintile. The simulation of future outcomes comes with inherent uncertainty, no matter the time horizon, but we attempted to provide a range of scenario analyses to capture how results might be impacted by key assumptions, including alternative time horizons. While we conducted a scenario analysis projecting the Gini coefficient and the associated relative income distribution over time, Gini projections are patchy and inconsistent, which limits our ability to vary the income distribution effectively. This approach also implicitly assumed that mean incomes and OOP costs would evolve relatively similarly over time, which ultimately did not impact the estimation of CHE. Projecting the state of health system financing (as well as the share of OOP vs public financing) in Ethiopia would further rely on political economy factors that are difficult to foresee. These projections also did not take into consideration the impact of the COVID-19 pandemic, which may result in large increases to the world’s poor (World Bank, 2021). Vaccine efficacy against high-risk HPV types other than HPV-16/18 (i.e. cross-protection) was also not included; we did not examine CC screening programmes and assumed that any ongoing screening programmes did not change as HPV vaccination introduction and delivery changed (which could have a significant impact on the number of CHE cases averted by HPV vaccination, as noted above with the care-seeking scenario analyses). Finally, as we relied on a static cohort model in this analysis, we were only able to estimate direct effects for vaccinated women, which excluded additional indirect benefits from herd immunity for unvaccinated women. Moreover, given the limited data on the burden of other HPV-related diseases in low- and middle-income countries, we did not evaluate the impact HPV vaccination may have on non-CCs in women and men, which likely would increase the value of all HPV vaccination strategies. There remains substantial uncertainty concerning the long-term impacts of HPV vaccination on CC incidence and mortality, but given our focus on key scenario assumptions affecting structural uncertainty and FRP benefits, additional uncertainty analysis of parameters jointly is largely left for future work.

The results of our work regarding the potential equity-improving impact of HPV vaccination are consistent with previous work on the equity impact of vaccines (Chang *et al*., 2018a,b; Riumallo-Herl *et al.*, 2018). The potential for HPV vaccination to provide FRP to the poorest households is also consistent with a previous study on routine HPV vaccination in China (Levin *et al.*, 2015). This further underscores the need for additional metrics beyond traditional CEA in order to account for the broader value of vaccination (Barnighausen *et al.*, 2014; Bloom *et al.*, 2018). Complementary analyses such as this one that address the potentially large equity impacts of policies can support decision-makers evaluating public health interventions.

## Conclusion

In summary, our approach incorporates equity dimensions into the economic evaluation of routine HPV vaccination in Ethiopia, a resource-constrained setting with currently low CC screening coverage. The pro-poor results of this study emphasize the continued priority of routine HPV vaccination once the global vaccine shortage is overcome. This analysis can help make decisions regarding routine HPV vaccination, including how to progress towards CC elimination (World Health Organization, 2019) both efficiently and equitably. The results can inform fair and efficient priority settings in low- and middle-income countries with similar demographic and economic characteristics.

## Supplementary Material

czab052_Supp

## Data Availability

The data underlying this article will be shared on reasonable request to the corresponding author.
