# Supplementary material for: Health gains and financial protection from human papillomavirus vaccination in Ethiopia: findings from a modelling study
Source: Health Policy Plan. 2021 May 4;36(6):891–9. doi: 10.1093/heapol/czab052 (PMC8227995; doi:10.1093/heapol/czab052)
Supplement: czab052_Supp [file czab052_supp.zip › Supplementary Appendix_v3.docx]

**Appendix**

**Appendix Table 1. National estimates of cervical cancer incidence by age group in Ethiopia (Memirie et al., 2018).**

| Age group  (years) | Cervical cancer incidence rate per 100,000 women |
| --- | --- |
| 0-4 | 0 |
| 5-9 | 0 |
| 10-14 | 0 |
| 15-19 | 4 |
| 20-24 | 4 |
| 25-29 | 4 |
| 30-34 | 4 |
| 35-39 | 4 |
| 40-44 | 33 |
| 45-49 | 43 |
| 50-54 | 78 |
| 55-59 | 78 |
| 60-64 | 81 |
| 65-69 | 69 |
| 70-74 | 70 |
| 75-79 | 43 |
| 80-84 | 43 |
| 85-99 | 43 |

**Appendix Table 2. Probabilistic sensitivity analysis (PSA) parameters and distributions**

| **Parameter** | **Probability distribution** | **Bound assumptions** | **Source** |
| --- | --- | --- | --- |
| HPV-16/18 type distribution | Beta-PERT | Bound width determined by regional minimum/maximum and regional average | (Guan et al., 2012) |
| Age-specific cervical cancer incidence | Beta-PERT | 95% CI = +/- 1.96x se = +/- 1.96x rate ÷ √cases | Inputs: Globocan 2020 (Ferlay et al.); Formula: (New York State Department of Health) |
| Stage distribution | Beta-PERT | Minimum and maximum as +/- 10% | WHO (Canfell et al., 2020) |
| Stage-specific 5-year survival & treatment access | Beta-PERT | Minimum and maximum as +/- 10% if percentage does not push >=1 or <=0 | WHO (Canfell et al., 2020) |

Note: As age-specific cervical cancer incidence is bounded by zero, the bound assumptions created the potential for asymmetrical uncertainty bounds.

**Appendix Table 3. Cases of catastrophic health expenditure (CHE) averted by routine two-dose human papillomavirus (HPV) vaccination scenario in Ethiopia: varying care-seeking in the population affected by cervical cancer (95% uncertainty intervals in parentheses).**

**(A) Assuming 2.4%**

| Scenario | Poorest | Poorer | Middle | Richer | Richest | Total |
| --- | --- | --- | --- | --- | --- | --- |
| 14yo Cohort, 95% Coverage | 50 | 10 | 10 | 30 | 0 | 100 |
|  | (40–50) | (10–10) | (10–10) | (20–30) |  | (80–110) |
| Routine, 50% Coverage Gradient | 2,300 | 1,200 | 1,000 | 2,430 | 0 | 6,930 |
|  | (1,800–2,400) | (920–1,230) | (760–1,020) | (1,850–2,490) |  | (5,330–7,140) |
| Routine, 50% Flat Coverage | 4,700 | 1,320 | 1,060 | 2,470 | 0 | 9,550 |
|  | (3,600–4,800) | (1,010–1,360) | (810–1,080) | (1,890–2,530) |  | (7,310–9,770) |

**(B) Assuming 50%**

| Scenario | Poorest | Poorer | Middle | Richer | Richest | Total |
| --- | --- | --- | --- | --- | --- | --- |
| 14yo Cohort, 95% Coverage | 1,090 | 310 | 240 | 570 | 0 | 2,210 |
|  | (830–1,120) | (230–320) | (190–250) | (440–590) |  | (1,690–2,280) |
| Routine, 50% Coverage Gradient | 48,100 | 25,000 | 20,800 | 50,600 | 0 | 145,000 |
|  | (36,700–49,300) | (19,100–25,600) | (15,900–21,300) | (38,600–51,800) |  | (110,000–148,000) |
| Routine, 50% Flat Coverage | 98,000 | 27,600 | 22,000 | 51,500 | 0 | 199,000 |
|  | (74,800–100,000) | (21,100–28,300) | (16,800–22,500) | (39,3000–52,800) |  | (152,000–204,000) |

Note: The findings assume a $482 OOP payment for cervical cancer treatment, which is considered a case of catastrophic health expenditure (CHE) for the poorest, poorer, middle, and richer consumption quintiles at a 40% threshold.

**Appendix Table 4. Cases of catastrophic health expenditure (CHE) averted by routine two-dose human papillomavirus (HPV) vaccination scenario in Ethiopia: assuming a linear gradient of cervical cancer incidence (95% uncertainty intervals in parentheses).**

| Scenario | Poorest | Poorer | Middle | Richer | Richest | Total |
| --- | --- | --- | --- | --- | --- | --- |
| 14yo Cohort, 95% Coverage | 200 | 190 | 100 | 130 | 0 | 620 |
|  | (100–200) | (150–200) | (80–100) | (100–140) |  | (430–640) |
| Routine, 50% Coverage Gradient | 10,600 | 19,200 | 10,200 | 14,500 | 0 | 54,500 |
|  | (8,100–10,900) | (14,600–19,600) | (7,810–10,500) | (11,100–14,900) |  | (41,600–55,900) |
| Routine, 50% Flat Coverage | 17,700 | 17,300 | 8,870 | 12,100 | 0 | 56,000 |
|  | (13,500–18,200) | (13,200–17,700) | (6,770–9,090) | (9,240–12,400) |  | (42,700–57,400) |

Note: The findings assume a $482 OOP payment for cervical cancer treatment, which is considered a case of catastrophic health expenditure (CHE) for the poorest, poorer, middle, and richer consumption quintiles at a 40% threshold.

**Appendix Table 5. Cases of catastrophic health expenditure (CHE) averted by** **routine two-dose human papillomavirus (HPV) vaccination scenario in Ethiopia: assuming no additional gradient of cervical cancer incidence (95% uncertainty intervals in parentheses).**

| Scenario | Poorest | Poorer | Middle | Richer | Richest | Total |
| --- | --- | --- | --- | --- | --- | --- |
| 14yo Cohort, 95% Coverage | 190 | 50 | 40 | 100 | 0 | 380 |
|  | (140–190) | (40–50) | (30–40) | (70–100) |  | (280–380) |
| Routine, 50% Coverage Gradient | 5,500 | 12,500 | 8,870 | 18,900 | 0 | 45,700 |
|  | (4,200–5,700) | (9,500–12,800) | (6,770–9,090) | (14,400–19,400) |  | (34,900–46,900) |
| Routine, 50% Flat Coverage | 10,600 | 13,000 | 8,870 | 18,200 | 0 | 50,600 |
|  | (8,100–10,900) | (9,900–13,300) | (6,770–9,090) | (13,900–18,600) |  | (38,600–51,900) |

Note: The findings assume a $482 OOP payment for cervical cancer treatment, which is considered a case of catastrophic health expenditure (CHE) for the poorest, poorer, middle, and richer consumption quintiles at a 40% threshold.

**Appendix Table 6.**

1. **Linear projections of Gini coefficient and out-of-pocket (OOP) payment levels overtime.**

| **Year** | **Gini** | **OOP** |
| --- | --- | --- |
| 1995* | 0.4460 | NA |
| 1999* | 0.3000 | NA |
| 2004* | 0.2980 | NA |
| 2010* | 0.3320 | NA |
| 2015* | 0.3500 | NA |
| 2019 | 0.3360 | 482 |
| 2020 | 0.3600 | 516 |
| 2021 | 0.3646 | 523 |
| 2022 | 0.3702 | 531 |
| 2023 | 0.3808 | 546 |
| 2024 | 0.3922 | 563 |
| 2025 | 0.3700 | 531 |
| 2026 | 0.3854 | 553 |
| 2027 | 0.3856 | 553 |
| 2028 | 0.3837 | 550 |
| 2029 | 0.3830 | 549 |
| 2030 | 0.3800 | 545 |
| 2031 | 0.3795 | 544 |
| 2032 | 0.3776 | 542 |
| 2033 | 0.3760 | 539 |
| 2034 | 0.3743 | 537 |
| 2035 | 0.3900 | 559 |
| 2036 | 0.3848 | 552 |
| 2037 | 0.3600 | 516 |
| 2038 | 0.3706 | 532 |
| 2039 | 0.3647 | 523 |
| 2040 | 0.3546 | 509 |
| 2041 | 0.3502 | 502 |
| 2042 | 0.3700 | 531 |
| 2043 | 0.3573 | 513 |
| 2044 | 0.3595 | 516 |
| 2045 | 0.3635 | 521 |
| 2046 | 0.3649 | 523 |
| 2047 | 0.3800 | 545 |
| 2048 | 0.3803 | 546 |
| 2049 | 0.3870 | 555 |
| 2050 | 0.3939 | 565 |
| 2051 | 0.4007 | 575 |
| 2052 | 0.3900 | 559 |
| 2053 | 0.4003 | 574 |
| 2054 | 0.4012 | 576 |
| 2055 | 0.4015 | 576 |
| 2056 | 0.4026 | 578 |
| 2057 | 0.4000 | 574 |
| 2058 | 0.4013 | 576 |
| 2059 | 0.3700 | 531 |
| 2060 | 0.3758 | 539 |
| 2061 | 0.3649 | 523 |
| 2062 | 0.3537 | 507 |
| 2063 | 0.3430 | 492 |
| 2064 | 0.3800 | 545 |
| 2065 | 0.3594 | 516 |
| 2066 | 0.3648 | 523 |
| 2067 | 0.3718 | 533 |
| 2068 | 0.3765 | 540 |
| 2069 | 0.3900 | 559 |
| 2070 | 0.3944 | 566 |
| 2071 | 0.4027 | 578 |
| 2072 | 0.4110 | 590 |
| 2073 | 0.4194 | 602 |
| 2074 | 0.4000 | 574 |
| 2075 | 0.4139 | 594 |
| 2076 | 0.4128 | 592 |
| 2077 | 0.4108 | 589 |
| 2078 | 0.4101 | 588 |
| 2079 | 0.4100 | 588 |
| 2080 | 0.4084 | 586 |
| 2081 | 0.3800 | 545 |
| 2082 | 0.3848 | 552 |
| 2083 | 0.3745 | 537 |
| 2084 | 0.3632 | 521 |
| 2085 | 0.3534 | 507 |
| 2086 | 0.3900 | 559 |
| 2087 | 0.3700 | 531 |
| 2088 | 0.3755 | 539 |
| 2089 | 0.3828 | 549 |
| 2090 | 0.3876 | 556 |
| 2091 | 0.4000 | 574 |
| 2092 | 0.4048 | 581 |
| 2093 | 0.4129 | 592 |
| 2094 | 0.4208 | 604 |
| 2095 | 0.4290 | 615 |
| 2096 | 0.4100 | 588 |
| 2097 | 0.4235 | 608 |
| 2098 | 0.4223 | 606 |
| 2099 | 0.4204 | 603 |
| 2100 | 0.4195 | 602 |
| 2101 | 0.4200 | 603 |
| 2102 | 0.4182 | 600 |
| 2103 | 0.3900 | 559 |
| 2104 | 0.3950 | 567 |
| 2105 | 0.3848 | 552 |
| 2106 | 0.3735 | 536 |
| 2107 | 0.3640 | 522 |
| 2108 | 0.4000 | 574 |
| 2109 | 0.3802 | 545 |
| 2110 | 0.3857 | 553 |
| 2111 | 0.3928 | 563 |
| 2112 | 0.3975 | 570 |
| 2113 | 0.4100 | 588 |
| 2114 | 0.4147 | 595 |
| 2115 | 0.4227 | 606 |
| 2116 | 0.4306 | 618 |
| 2117 | 0.4388 | 629 |
| 2118 | 0.4200 | 603 |

* Estimates of the Gini coefficient from the World Development Indicators (World Bank, 2019).

1. **Income distribution and catastrophic health expenditure (CHE) cutoff at a 40% threshold by wealth quintile following a lognormal distribution with part (A) Gini coefficient projections as the shape parameter.**

| **Year** | **Income** | | | | | **CHE Threshold Level** | | | | |
| --- | --- | --- | --- | --- | --- | --- | --- | --- | --- | --- |
|  | **Poorest** | **Poorer** | **Middle** | **Richer** | **Richest** | **Poorest** | **Poorer** | **Middle** | **Richer** | **Richest** |
| 2019 | 241 | 465 | 674 | 940 | 1,529 | 96 | 186 | 270 | 376 | NA |
| 2020 | 213 | 439 | 659 | 945 | 1,592 | 85 | 176 | 264 | 378 | NA |
| 2021 | 208 | 434 | 655 | 945 | 1,604 | 83 | 174 | 262 | 378 | NA |
| 2022 | 202 | 428 | 652 | 948 | 1,619 | 81 | 171 | 261 | 379 | NA |
| 2023 | 189 | 415 | 644 | 949 | 1,650 | 76 | 166 | 258 | 379 | NA |
| 2024 | 178 | 404 | 637 | 952 | 1,684 | 71 | 162 | 255 | 381 | NA |
| 2025 | 202 | 429 | 653 | 948 | 1,620 | 81 | 172 | 261 | 379 | NA |
| 2026 | 185 | 411 | 641 | 948 | 1,665 | 74 | 164 | 256 | 379 | NA |
| 2027 | 185 | 411 | 641 | 949 | 1,664 | 74 | 165 | 256 | 380 | NA |
| 2028 | 187 | 412 | 641 | 948 | 1,657 | 75 | 165 | 257 | 379 | NA |
| 2029 | 188 | 414 | 644 | 950 | 1,659 | 75 | 166 | 258 | 380 | NA |
| 2030 | 190 | 416 | 645 | 949 | 1,651 | 76 | 167 | 258 | 380 | NA |
| 2031 | 191 | 418 | 646 | 949 | 1,648 | 76 | 167 | 258 | 379 | NA |
| 2032 | 194 | 421 | 647 | 948 | 1,641 | 77 | 168 | 259 | 379 | NA |
| 2033 | 195 | 421 | 647 | 947 | 1,635 | 78 | 169 | 259 | 379 | NA |
| 2034 | 197 | 424 | 649 | 948 | 1,634 | 79 | 170 | 260 | 379 | NA |
| 2035 | 180 | 406 | 638 | 950 | 1,675 | 72 | 163 | 255 | 380 | NA |
| 2036 | 185 | 411 | 642 | 949 | 1,661 | 74 | 165 | 257 | 380 | NA |
| 2037 | 213 | 439 | 659 | 946 | 1,593 | 85 | 176 | 264 | 378 | NA |
| 2038 | 201 | 429 | 653 | 948 | 1,623 | 81 | 171 | 261 | 379 | NA |
| 2039 | 208 | 435 | 656 | 946 | 1,606 | 83 | 174 | 262 | 378 | NA |
| 2040 | 220 | 446 | 662 | 944 | 1,579 | 88 | 178 | 265 | 378 | NA |
| 2041 | 224 | 451 | 665 | 943 | 1,569 | 90 | 180 | 266 | 377 | NA |
| 2042 | 203 | 429 | 653 | 948 | 1,620 | 81 | 172 | 261 | 379 | NA |
| 2043 | 216 | 443 | 660 | 945 | 1,585 | 86 | 177 | 264 | 378 | NA |
| 2044 | 214 | 440 | 660 | 946 | 1,592 | 85 | 176 | 264 | 379 | NA |
| 2045 | 209 | 435 | 656 | 946 | 1,601 | 84 | 174 | 263 | 378 | NA |
| 2046 | 208 | 435 | 657 | 947 | 1,607 | 83 | 174 | 263 | 379 | NA |
| 2047 | 191 | 417 | 645 | 949 | 1,646 | 76 | 167 | 258 | 379 | NA |
| 2048 | 191 | 417 | 645 | 949 | 1,649 | 76 | 167 | 258 | 380 | NA |
| 2049 | 184 | 409 | 640 | 948 | 1,666 | 73 | 164 | 256 | 379 | NA |
| 2050 | 176 | 401 | 635 | 952 | 1,690 | 70 | 161 | 254 | 381 | NA |
| 2051 | 169 | 394 | 630 | 951 | 1,707 | 68 | 158 | 252 | 380 | NA |
| 2052 | 180 | 406 | 638 | 950 | 1,677 | 72 | 162 | 255 | 380 | NA |
| 2053 | 169 | 394 | 631 | 951 | 1,708 | 68 | 158 | 252 | 381 | NA |
| 2054 | 168 | 393 | 630 | 951 | 1,708 | 67 | 157 | 252 | 381 | NA |
| 2055 | 168 | 392 | 629 | 950 | 1,709 | 67 | 157 | 251 | 380 | NA |
| 2056 | 168 | 392 | 629 | 950 | 1,714 | 67 | 157 | 251 | 380 | NA |
| 2057 | 170 | 394 | 631 | 950 | 1,705 | 68 | 158 | 252 | 380 | NA |
| 2058 | 169 | 393 | 629 | 951 | 1,708 | 67 | 157 | 252 | 380 | NA |
| 2059 | 202 | 428 | 652 | 947 | 1,619 | 81 | 171 | 261 | 379 | NA |
| 2060 | 195 | 422 | 648 | 948 | 1,638 | 78 | 169 | 259 | 379 | NA |
| 2061 | 207 | 434 | 656 | 946 | 1,606 | 83 | 173 | 262 | 378 | NA |
| 2062 | 220 | 446 | 662 | 943 | 1,574 | 88 | 178 | 265 | 377 | NA |
| 2063 | 233 | 458 | 670 | 942 | 1,546 | 93 | 183 | 268 | 377 | NA |
| 2064 | 191 | 417 | 644 | 948 | 1,648 | 76 | 167 | 258 | 379 | NA |
| 2065 | 214 | 440 | 660 | 946 | 1,592 | 85 | 176 | 264 | 378 | NA |
| 2066 | 207 | 434 | 654 | 944 | 1,606 | 83 | 173 | 262 | 378 | NA |
| 2067 | 200 | 426 | 651 | 947 | 1,626 | 80 | 171 | 261 | 379 | NA |
| 2068 | 195 | 421 | 648 | 949 | 1,640 | 78 | 168 | 259 | 380 | NA |
| 2069 | 180 | 406 | 638 | 950 | 1,675 | 72 | 162 | 255 | 380 | NA |
| 2070 | 176 | 401 | 634 | 950 | 1,688 | 70 | 160 | 254 | 380 | NA |
| 2071 | 167 | 391 | 628 | 950 | 1,712 | 67 | 156 | 251 | 380 | NA |
| 2072 | 159 | 382 | 622 | 951 | 1,735 | 63 | 153 | 249 | 381 | NA |
| 2073 | 150 | 372 | 615 | 951 | 1,759 | 60 | 149 | 246 | 380 | NA |
| 2074 | 170 | 394 | 630 | 951 | 1,707 | 68 | 158 | 252 | 380 | NA |
| 2075 | 156 | 379 | 620 | 953 | 1,749 | 62 | 152 | 248 | 381 | NA |
| 2076 | 157 | 380 | 620 | 951 | 1,742 | 63 | 152 | 248 | 380 | NA |
| 2077 | 159 | 382 | 621 | 950 | 1,734 | 64 | 153 | 249 | 380 | NA |
| 2078 | 159 | 383 | 623 | 951 | 1,733 | 64 | 153 | 249 | 381 | NA |
| 2079 | 160 | 383 | 623 | 953 | 1,735 | 64 | 153 | 249 | 381 | NA |
| 2080 | 161 | 385 | 624 | 950 | 1,730 | 65 | 154 | 250 | 380 | NA |
| 2081 | 191 | 417 | 645 | 949 | 1,648 | 76 | 167 | 258 | 379 | NA |
| 2082 | 186 | 413 | 642 | 949 | 1,662 | 74 | 165 | 257 | 380 | NA |
| 2083 | 197 | 423 | 649 | 949 | 1,634 | 79 | 169 | 260 | 379 | NA |
| 2084 | 210 | 436 | 657 | 947 | 1,602 | 84 | 174 | 263 | 379 | NA |
| 2085 | 221 | 447 | 663 | 944 | 1,576 | 88 | 179 | 265 | 378 | NA |
| 2086 | 180 | 406 | 638 | 950 | 1,678 | 72 | 162 | 255 | 380 | NA |
| 2087 | 202 | 429 | 652 | 946 | 1,620 | 81 | 171 | 261 | 379 | NA |
| 2088 | 196 | 423 | 649 | 948 | 1,637 | 78 | 169 | 259 | 379 | NA |
| 2089 | 188 | 413 | 642 | 948 | 1,655 | 75 | 165 | 257 | 379 | NA |
| 2090 | 183 | 408 | 640 | 950 | 1,669 | 73 | 163 | 256 | 380 | NA |
| 2091 | 170 | 395 | 631 | 951 | 1,708 | 68 | 158 | 252 | 380 | NA |
| 2092 | 165 | 389 | 627 | 952 | 1,718 | 66 | 156 | 251 | 381 | NA |
| 2093 | 157 | 379 | 619 | 950 | 1,740 | 63 | 152 | 248 | 380 | NA |
| 2094 | 150 | 372 | 615 | 952 | 1,765 | 60 | 149 | 246 | 381 | NA |
| 2095 | 142 | 361 | 606 | 950 | 1,788 | 57 | 144 | 242 | 380 | NA |
| 2096 | 160 | 383 | 623 | 950 | 1,731 | 64 | 153 | 249 | 380 | NA |
| 2097 | 147 | 368 | 611 | 951 | 1,773 | 59 | 147 | 245 | 381 | NA |
| 2098 | 148 | 369 | 612 | 951 | 1,769 | 59 | 148 | 245 | 380 | NA |
| 2099 | 149 | 371 | 614 | 952 | 1,763 | 60 | 148 | 246 | 381 | NA |
| 2100 | 151 | 373 | 615 | 951 | 1,761 | 60 | 149 | 246 | 381 | NA |
| 2101 | 150 | 372 | 615 | 952 | 1,763 | 60 | 149 | 246 | 381 | NA |
| 2102 | 151 | 374 | 616 | 952 | 1,758 | 61 | 149 | 246 | 381 | NA |
| 2103 | 180 | 405 | 637 | 948 | 1,675 | 72 | 162 | 255 | 379 | NA |
| 2104 | 175 | 400 | 634 | 949 | 1,689 | 70 | 160 | 253 | 380 | NA |
| 2105 | 186 | 412 | 642 | 948 | 1,663 | 74 | 165 | 257 | 379 | NA |
| 2106 | 198 | 424 | 650 | 947 | 1,630 | 79 | 170 | 260 | 379 | NA |
| 2107 | 209 | 435 | 656 | 946 | 1,605 | 83 | 174 | 263 | 378 | NA |
| 2108 | 170 | 394 | 630 | 950 | 1,705 | 68 | 157 | 252 | 380 | NA |
| 2109 | 191 | 417 | 645 | 949 | 1,651 | 76 | 167 | 258 | 380 | NA |
| 2110 | 185 | 411 | 641 | 949 | 1,664 | 74 | 164 | 257 | 380 | NA |
| 2111 | 178 | 403 | 637 | 951 | 1,683 | 71 | 161 | 255 | 380 | NA |
| 2112 | 172 | 397 | 631 | 949 | 1,696 | 69 | 159 | 253 | 380 | NA |
| 2113 | 159 | 383 | 623 | 951 | 1,735 | 64 | 153 | 249 | 380 | NA |
| 2114 | 155 | 378 | 618 | 950 | 1,744 | 62 | 151 | 247 | 380 | NA |
| 2115 | 148 | 369 | 612 | 951 | 1,772 | 59 | 147 | 245 | 381 | NA |
| 2116 | 139 | 359 | 605 | 951 | 1,791 | 56 | 144 | 242 | 380 | NA |
| 2117 | 132 | 350 | 598 | 951 | 1,818 | 53 | 140 | 239 | 381 | NA |
| 2118 | 150 | 372 | 615 | 952 | 1,761 | 60 | 149 | 246 | 381 | NA |

**Appendix Table 7. Cases of catastrophic health expenditure (CHE) averted by routine two-dose human papillomavirus (HPV) vaccination scenario in Ethiopia: varying time horizon (95% uncertainty intervals in parentheses).**

1. **10 years**

| Scenario | Poorest | Poorer | Middle | Richer | Richest | Total |
| --- | --- | --- | --- | --- | --- | --- |
| 14yo Cohort, 95% Coverage | 5 | 1 | 1 | 3 | 0 | 10 |
|  | (4–5) | (1–1) | (1–1) | (2–3) |  | (7–11) |
| Routine, 50% Coverage Gradient | 8 | 4 | 4 | 9 | 0 | 25 |
|  | (6–8) | (3–5) | (3–4) | (6–10) |  | (18–27) |
| Routine, 50% Flat Coverage | 17 | 5 | 4 | 9 | 0 | 35 |
|  | (12–19) | (4–4) | (3–4) | (7–10) |  | (25–38) |

1. **25 years**

| Scenario | Poorest | Poorer | Middle | Richer | Richest | Total |
| --- | --- | --- | --- | --- | --- | --- |
| 14yo Cohort, 95% Coverage | 15 | 4 | 3 | 8 | 0 | 30 |
|  | (11–16) | (3–4) | (2–3) | (6–8) |  | (22–32) |
| Routine, 50% Coverage Gradient | 61 | 32 | 26 | 64 | 0 | 183 |
|  | (45–65) | (23–34) | (19–38) | (47–68) |  | (135–194) |
| Routine, 50% Flat Coverage | 124 | 35 | 28 | 65 | 0 | 252 |
|  | (92–132) | (26–37) | (21–30) | (48–69) |  | (186–268) |

1. **50 years**

| Scenario | Poorest | Poorer | Middle | Richer | Richest | Total |
| --- | --- | --- | --- | --- | --- | --- |
| 14yo Cohort, 95% Coverage | 74 | 21 | 17 | 39 | 0 | 150 |
|  | (56–77) | (16–22) | (13–17) | (29–40) |  | (114–156) |
| Routine, 50% Coverage Gradient | 943 | 490 | 407 | 992 | 0 | 2,833 |
|  | (717–971) | (373–505) | (310–420) | (754–1,021) |  | (2,153–2,916) |
| Routine, 50% Flat Coverage | 1,920 | 541 | 431 | 1,010 | 0 | 3,902 |
|  | (1,460–1,977) | (411–557) | (328–444) | (767–1,040) |  | (2,966–4,018) |

Note: The findings assume a $482 OOP payment for cervical cancer treatment, which is considered a case of catastrophic health expenditure (CHE) for the poorest, poorer, middle, and richer consumption quintiles at a 40% threshold. The findings are presented without rounding to differentiate between scenarios, but this is not intended to reflect additional certainty relative to primary results.

**References**

CANFELL, K., KIM, J. J., BRISSON, M., KEANE, A., SIMMS, K. T., CARUANA, M., BURGER, E. A., MARTIN, D., NGUYEN, D. T. N., BÉNARD, É., SY, S., REGAN, C., DROLET, M., GINGRAS, G., LAPRISE, J. F., TORODE, J., SMITH, M. A., FIDAROVA, E., TRAPANI, D., BRAY, F., ILBAWI, A., BROUTET, N. & HUTUBESSY, R. 2020. Mortality impact of achieving WHO cervical cancer elimination targets: a comparative modelling analysis in 78 low-income and lower-middle-income countries. *Lancet,* 395**,** 591-603.

FERLAY, J., COLOMBET, M. & BRAY, F. GLOBOCAN 2020. Age-specific cancer cases. IARC Cancer Base No. 9. Lyon, France: International Agency for Research on Cancer; 2018. Available at: <http://globocan.iarc.fr/Pages/age-specific_table_sel.aspx>.

GUAN, P., HOWELL-JONES, R., LI, N., BRUNI, L., DE SANJOSÉ, S., FRANCESCHI, S. & CLIFFORD, G. M. 2012. Human papillomavirus types in 115,789 HPV-positive women: A meta-analysis from cervical infection to cancer. *Int J Cancer,* 131**,** 2349-2359.

MEMIRIE, S. T., HABTEMARIAM, M. K., ASEFA, M., DERESSA, B. T., ABAYNEH, G., TSEGAYE, B., ABRAHA, M. W., ABABI, G., JEMAL, A., REBBECK, T. R. & VERGUET, S. 2018. Estimates of Cancer Incidence in Ethiopia in 2015 Using Population-Based Registry Data. *Journal of Global Oncology,* 4**,** 1-11.

NEW YORK STATE DEPARTMENT OF HEALTH About Age Adjusted Rates, 95% Confidence Intervals and Unstable Rates. New York State Cancer Registry. March 2018. Available at: <https://www.health.ny.gov/statistics/cancer/registry/age.htm>.

WORLD BANK 2019. World development indicators. Washington, DC: The World Bank; 2019. Last updated: 16 December 2020. [Online] Accessed 14 February 2021. Available at: <http://data.worldbank.org/>.
